# Prediction of First-Order Phase Transition with Electron–Phonon Interaction

**DOI:** 10.1021/acs.jpcc.4c00958

**Published:** 2024-06-11

**Authors:** Mario Graml, Kurt Hingerl

**Affiliations:** † Center for Surface- and Nanoanalytics, Johannes Kepler Universität, Altenbergerstr. 69, A-4040 Linz, Austria; ‡ School of Education, Johannes Kepler Universität, Altenbergerstr. 69, A-4040 Linz, Austria

## Abstract

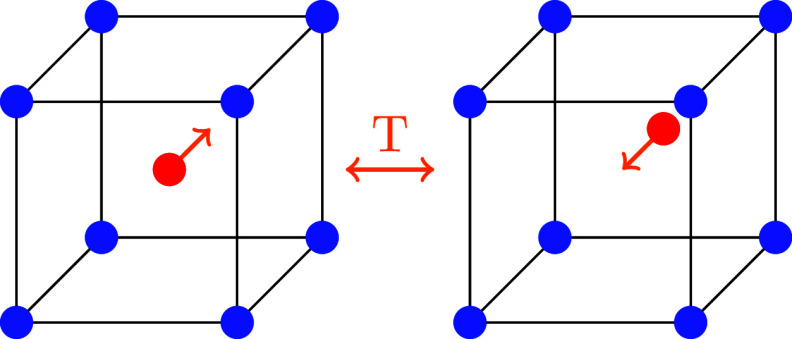

Phase transitions in solids occur due to the shifting balance between the binding energies and entropic contributions of different crystal structures, even though the underlying Hamiltonian remains the same. This work demonstrates that incorporating electron–phonon interactions in the Hamiltonian results in distinct free energies at different temperatures, thus leading to a first-order phase transition. Contrary to prior investigations, taking into consideration the quantum mechanical kinetic energy operator of the nucleus by employing Bogoliubov’s inequality yields a first-order phase transition. An equation is implicitly derived to determine the critical temperature of the first-order phase transition. Furthermore, an estimation is made to evaluate the latent heat and the resulting positional displacement of the nucleus. Comparing the present findings with previous ones allows setting parameter boundaries for both first- and second-order phase transitions.

## 1 Introduction

Comprehensive theories for first-order phase transitions are lacking because at the phase transition temperature (PTT), the most extensive quantities exhibit discontinuous behavior. The Gibbs energy, which has only intensive derivatives, remains continuous. However, its first derivative at the PTT is nonanalytical and discontinuous. The same reasoning can be applied to the Helmholtz free energy *F* for small changes in volume *V* at the PTT. Additionally, the free energy is directly related to statistics through the partition function. Structural phase transitions are usually first-order ones. These, for example, solid–solid phase transitions between different crystal structures occur because the balance between binding energies and entropic contributions shifts as a function of temperature. This review addresses conceptual questions regarding the general modeling of first-order solid–solid phase transitions.

One initial inquiry is why the same ab initio Hamiltonian is applicable to both the high and low temperature phases (HTP/LTP), as well as possibly other phases. Is there a way to determine, based on a single ab initio Hamiltonian, distinct scenarios that display behavior similar to the first-order phase transition observed in experiments?

The electronic configuration primarily determines the binding energies in both phases of a solid, while the entropic contributions are mainly due to low phonon frequencies.^1,2^ In the case of first-order phase transitions, both quantities exhibit a jump at the PTT. When electrons (or quasiparticles in density functional theory) and phonons do not interact, the partition sum can be factorized, and the free energy for both can simply be added. Therefore, in the scenario where electrons and phonons are separated, the phase with a lower binding energy but a denser phononic energy spectrum leads to a significant increase in entropy density.
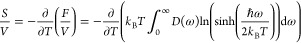
1This increase in the entropy density drives the PT to the HTP when the temperature *T* is enhanced. The frequency-dependent phonon density of states is denoted by *D*(ω) and the Boltzmann constant is *k*_B_. If a particular phase transition is being modeled, the experimentally measured (or estimated) crystal structure can be utilized with density functional theory to ascertain the binding energy and phonon dispersion curves. In order to identify solid–solid phase transitions, it is necessary to calculate binding energies and entropies for the relevant space groups (out of a total of 230) and select the option with the lowest value of *F*. Nonetheless, this separated approach leaves the question unresolved, which microscopic term in the Hamiltonian initiates the phase transition.

## 2 Model Hamiltonian

We tackle this issue by modeling first-order phase transitions (PTs) without specifying any crystal structure. This is achieved by introducing the electron–phonon interaction (EPI) as a third term in the electron–phonon Hamiltonian, as shown in eq 4. In contrast to quantum mechanical perturbation theory, the free energy is calculated by taking the trace of all eigenenergies *E*_*j*_ of the Hamiltonian operator including those of excited states. If these eigenenergies are known, the free energy can be written as
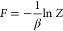
2
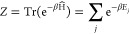
3with β = (*k*_B_*T*)^−1^.

To answer these questions, we use a simple model Hamiltonian, assuming that only a single nucleus will be displaced. This Hamiltonian has been previously derived by Kristoffel and Konsel^3−6^ from a more general EPI Hamiltonian. We calculate *F* additionally by incorporating the kinetic energy operator as well as the EPI, in contrast to the aforementioned works.
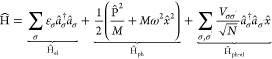
4The Hamiltonian consists of an electronic part Ĥ_el_ with σ labeling the bands (in our case, only two dispersionless, valence and conduction band), a phononic part Ĥ_ph_ and the EPI term Ĥ_ph-el_. The mass of the nucleus *M* oscillates with the frequency ω. The numbers of unit cells in the crystal represented by *N* and *V*_σσ*′*_ are the linear coupling coefficients of the EPI. Already Ginzburg, Anderson, and Cochran^7−10^ proposed such a Hamiltonian; their idea was taken up by Kristoffel and Konsel, proposing an EPI Hamiltonian correlating the shift of the nucleus to electron operators. By coupling electron and lattice dynamics through EPI, they found a nonanalyticity of the derivatives of *F* showing the occurrence of a PT. However, their approach only led to a second-order transition (continuous shift of the nucleus) because the resulting *F* turned out to be once differentiable. The approximation employed by Kristoffel and Konsel neglected the kinetic energy of the nucleus.

This model Hamiltonian can be solved precisely in the classical high temperature limit, as outlined in the appendix. An exact QM solution is not possible when adding EPI because the eigenenergies *E*_*j*_ depend on the nuclei and electronic coordinates. Even without solving the problem analytically, we can discuss the two limiting cases of strong and weak EPI. If the EPI term is huge, new “quasiparticles” will form with a strong entanglement between electrons and nuclei (in our case, one nucleus). At an increased temperature, considering a more realistic model with phonon–phonon interaction, the entanglement will weaken compared to multi phonon scattering processes, which are neglected in our model Hamilton, and the nucleus motion as well as electrons can be described independently.

Conversely, for moderate EPI, the concept of electronic band structure persists at low temperatures with perturbative corrections. As the temperature rises, electronic energies are less affected compared to the contribution of phonons, as the number of phonons increases and the related entropy is enhanced. To model this first-order transition from the low temperature phase (LTP) to the high temperature phase (HTP), we compute the QM free energy using *two* Bogoliubov inequalities,^11,12^ stemming from the *same* Hamiltonian. By employing this technique, we obtain accurate values of the free energy in the limiting cases β approaching either zero or infinity. Subsequently, by applying the aforementioned inequality, both asymptotic behaviors of the free energy are captured.

For weak EPI, consistent with the discussion in ref (14), the “LTP” is identified as the phase where the EPI term can be neglected. Conversely, the “HTP” is the one where the EPI contributions dominate over the electronic energy, owing to the high average occupation number of phonons. As shown in ref (15), the best approximation for an upper limit of the free energy *F* for both phases is obtained by evaluating the second Bogoliubov inequality. This method provides an approximate calculation for the system’s free energy in both limiting cases. Therefore, the right hand side of eq 5a approximates the free energy for the LTP well, and the right hand side of eq 5b approximates the free energy for the HTP.


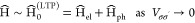
5a
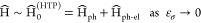
5b

The Hamiltonians Ĥ_0_^(LTP)^ and Ĥ_0_^(HTP)^ are both analytically solvable, and the unperturbed free energies *F*_0_^(LTP/HTP)^ can be computed. Then, the second Bogoliubov inequality^11^ is used to compute the LTP free energy *F*^(LTP)^ and the HTP free energy *F*^(HTP)^, always treating the previously neglected term as perturbation. The physical system realizes the phase with the lower of the two free energies, which are modeled as *F*_Mod_^(LTP)^ and *F*_Mod_^(HTP)^, using the same criteria for the PT.

We start by evaluating the LTP with vanishing EPI. The Schrödinger equation for the Hamiltonian Ĥ_0_^(LTP)^
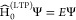
6is a standard problem. The electronic Hamiltonian *H*_el_ and the phononic Hamiltonian Ĥ_ph_ commute, resulting in two independent subsystems. We find the energy
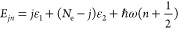
7awhere *j* represents the number of excited electrons in the conduction band, *N*_e_ represents the number of affected electrons in the Brillouin zone, and *n* represents the state of the phonon. Realistically, all electrons with any  and all nuclei are exposed to EPI. Experimentally, *one* ion in the unit cell is shifted, which is in the model connected to a certain number of electrons, labeled *N*_e_. The unperturbed Helmholtz energy *F*_0_^(LTP)^ is calculated with eq 7a, including all combinatorial possibilities, using the factorization of the subsystems, as
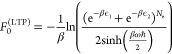
8The perturbed Helmholtz energy is evaluated using Bogoliubov’s inequality, expressed as
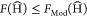
9where
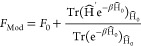
10This inequality (eq 9) asserts that the true Helmholtz energy of the system does not exceed the modeled Helmholtz energy calculated according to eq 10. Here, in eq 10, the correction term is computed through the expectation value of the perturbation Hamiltonian Ĥ*′* with respect to the eigenfunctions of the unperturbed Hamiltonian Ĥ_0_.

The term
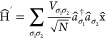
11does not change the model free energy *F*_Mod_^(LTP)^ in first-order because it is antisymmetric in x̂, yielding
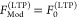
12In addition, the electronic contribution results in the same formula because the expectation value of the term ⟨*â*_σ_^†^_1_*â*_σ_2__ ⟩ with σ_1_≠ σ_2_ vanishes. We note in passing that this has already been pointed out in ref (16). K. Nasu then came to the conclusion that it is necessary to calculate higher-order perturbation terms for a QM perturbation result. However, the second Bogoliubov inequality for *F* can be proven for arbitrary strength of the perturbation^17^ and therefore does not require the calculation of higher perturbation orders.

Next, we turn to the HTP Hamiltonian Ĥ_0_^(HTP)^:
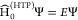
13a
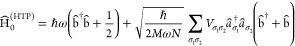
13b
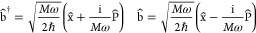
13c
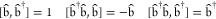
13d

In the second line, Ĥ_0_^(HTP)^ is rewritten using the ladder operators , defined by eq (13) with the commutation properties of bosons in eq 13d. We then proceed by shifting the original ladder operators, labeled by a bar (b̅̂^†^, b̅̂) (eq 14a), which decouples the b̅̂^†^, b̅̂ and *â*^†^, *â* operators in eq 14c.
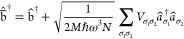
14a
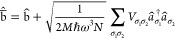
14b
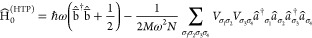
14c
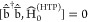
14d
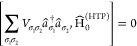
14e
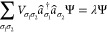
14f

Ĥ_0_^(HTP)^ is commutated with b̅̂^†^b̅̂ and represents a shifted harmonic oscillator. Due to the commutation relation given by eq 14e with the EPI potential, the eigenvalue equation in eq 14f results with eigenvalues λ. Equation 14a suggests that the barred operators are modified phonon operators influenced by the EPI potential. The eigenvalue problem in eq 14f is solved with λ = ± *V*. The respective eigenfunctions for the electronic bands in a two band model are renormalized due to EPI to .

The literature, such as ref (18), provides well-known solutions for the shifted harmonic oscillator in eq 14c. These solutions can be expressed as follows:

For the energy levels, we have
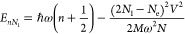
15

The corresponding eigenfunctions are given by
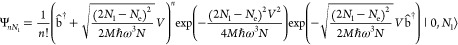
16

Here, |*N*_1_⟩ represents the state
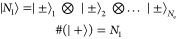
17where the nomenclature indicates that out of *N*_e_ electrons, *N*_1_ electrons are in the |+⟩ state. The expectation value of the *discrete* displacement of the nucleus ⟨*x*⟩_*nN*_1__ depends on the *n*th phonon mode and the number of electrons *N*_1_ in the lower band and is calculated as
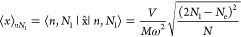
18The total displacement of the nucleus is then calculated as
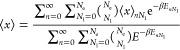
19*F*_0_^(HTP)^ is calculated as
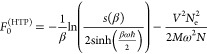
20with
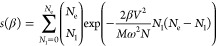
21Using Bogoliubov’s second inequality with the perturbation
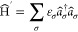
22one finds for *F*_Mod_^(HTP)^
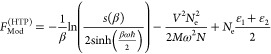
23In the appendix, it is shown that eq 23 derived through Bogoliubov’s approach, which corresponds to the classical free energy in the high temperature limit β → 0. Our focus is solely on the high temperature behavior; consequently, we replace *s*(β) with its value at β = 0, which is *s*(0) = 2^*N*_e_^.

## 3 Prediction of the First-Order Phase Transition

**Figure 1:**
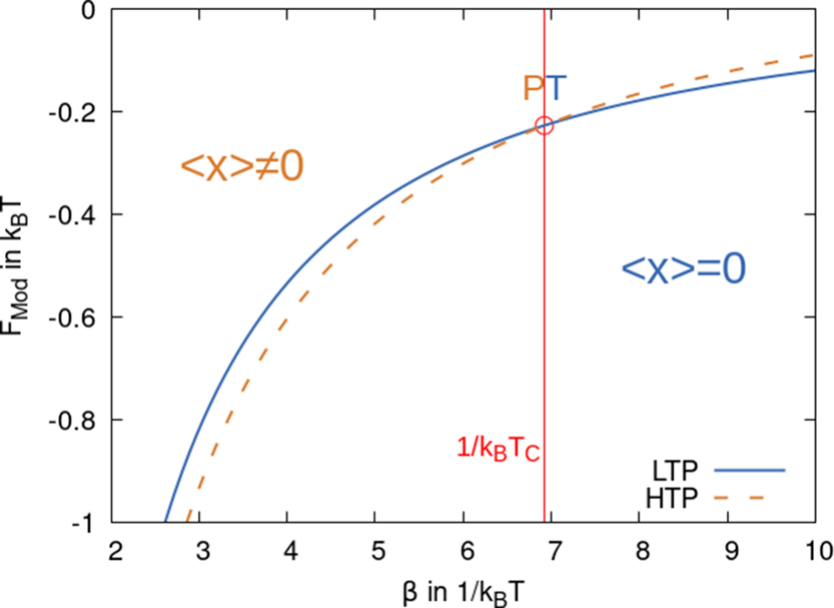
Free energy of the HTP and the LTP is plotted as a function of the inverse temperature β for the chosen parameters in arb. units: ε_1_ = 0, ε_2_ = 1, *N*_e_ = 1, *N* = 1, *ℏ*ω = 0.03, and *V*^2^/(2*M*ω^2^) = 0.4. Solid (blue) line represents the LTP, and the dashed (orange) line represents the HTP, respectively. Red vertical line indicates the critical inverse temperature at which the PT occurs.

Both model free energies *F*_Mod_^(LTP)^ as well as *F*_Mod_^(HTP)^ allow us to predict the PTT and validate the order of the phase transition: In Figure 1, we plot as a function of the inverse temperature the two model free energies. For small, β*F*_Mod_^(HTP)^ is below *F*_Mod_^(LTP)^, and so, the HTP is realized at high temperature. With both curves one can determine the intersection yielding the inverse PTT. The derivatives of the free energies with respect to temperature at the PTT are different, and a first-order PT results.

This first-order phase transition is accompanied by a jump in the vectorial order parameter, defined as the expectation value of the displacement of the nucleus.

**Figure 2:**
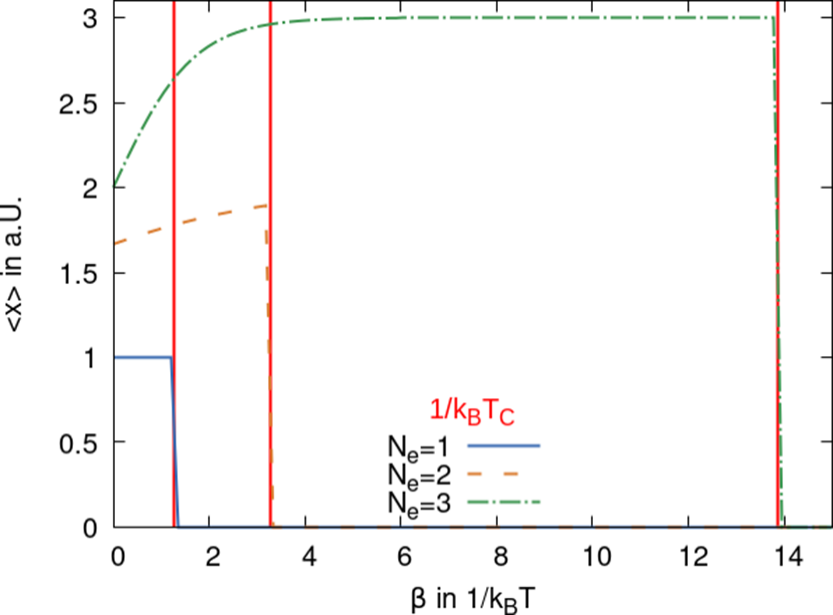
Plot shows the order parameter for a different electron number per unit cell as a function of the inverse temperature β, for the chosen parameters in arbitrary units: *N* = 1, *V*^2^/(2*M*ω^2^) = 0.15, and *V*/(*M*ω^2^) = 1. Solid (blue) line represents the one-electron case, the dashed (orange) line represents the two-electron case, and the dotted (green) line represents the three-electron case. Red vertical line indicates the critical inverse temperature at which the PT occurs.


As shown in Figure 2, the total displacement of the nucleus, calculated with eq 19, increases with the number of electrons but is always discontinuous with *T*_C_ dependent on temperature. For a large number of electrons, there are more configurations than for a smaller number, resulting in a more complex mixture of configurations. An implicit equation for the inverse Curie temperature β_C_ can be derived, assuming a bandgap Δ = ε_2_ – ε_1_
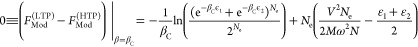
24
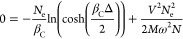
25The first term of eq 25 is bounded by
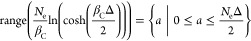
26yielding a condition for the PT:
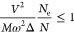
27Only a comparable **moderate** electron–phonon coupling *V* induces a PT, despite the discrete shift of the nucleus (eq 18) scales linear with *V*. For small and large band gap semiconductors, respectively (eq 25), the inverse PTT can be expressed as

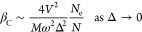
28a
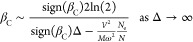
28b

Analytically, the order of the PT is determined by evaluating the first derivative
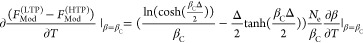
29Comparing the first and second contribution on the rhs of eq 29 and using , one confirms that the PT represented in eq 25 is indeed, always of the first order. For both limiting cases, an increase of the electron number enhances the electron–phonon coupling and the switch to the HTP occurs at lower temperature.

Using the analytical expression for *F*, the latent heat, defined as *δ L* = *δ S T*_C_ with the entropy difference *δ S* = *S*_HTP_ – *S*_LTP_ at the PT point, can be directly calculated as
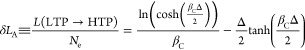
30A discussion of this result, together with eqs 26 and 28a, shows that the latent heat increases for systems with (a) the electron–phonon coupling constant *V*, (b) small nucleus mass *M*, (c) low frequency ω, (d) a comparatively high electron number per unit cell *N*_e_/*N*, and (e) large bandgaps.

We now return to the two cases of strong and weak or moderate EPI and combine our results (eq 27) with those by Kristoffel and Konsel. They computed the free energy neglecting the QM nature of the nucleus motion.^4^ The recalculation of their results for the case of variable electron number yields
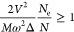
As mentioned before, their derivation provides a criterion for a PT of second order. Combining these two conditions, *that is, assuming that their approximation as well as the Bogoliubov approach are valid in the respective parameter ranges*, yields
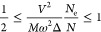
31This double inequality shows that both orders of phase transitions can happen if the electron–phonon coupling has the appropriate strength. Below the left bound of 1/2, the resulting PT is of first order; above the right bound of 1, a PT of second order occurs. In the parameter range between 1/2 and 1, their combination yields a contradictory (i.e., no definite) PT order.

As discussed before, this behavior is related to the relative size of the EPI:

For a strongly correlated system (strong electron–phonon coupling) even at a low temperature, the formed electron–nucleus pair, termed polaron by Landau, is stable and energetically dominates, so enhancing temperature mainly affects the Fermionic system resulting in a second-order PT with continuous nucleus (nuclei) shift. This effect occurs in highly polarizable systems with a large Fröhlich coupling constant α.^19^
Conversely, a first-order PT with latent heat can occur if the system’s ground state is not polaronic, which is the case for small Fröhlich coupling constants. Due to the small number and low frequency of phonons at low temperature, the EPI contribution to *F* is negligible. However, as the temperature increases, this term becomes significant due to the increase in the number of phonons and the excitation of low-frequency phonons that couple the nucleus and the electrons.

In order to validate our model, we apply it to BaTiO_3_, utilizing crystal parameters taken from the Materials Project.^20^ We will estimate the PTT for the tabulated tetragonal-cubic phase transition with a small band gap change from 1.83 eV in the cubic phase to 2.26 eV in the tetragonal phase, both systems realizing that direct band gaps are at the Γ point. Similarly, the phonon spectrum exhibits significant changes mainly at the transverse optical (TO) mode (ω = 1.56 × 10^13^ s^–1^) during the tetragonal-cubic phase transition, as reported in Zhong.^21^

In order to maintain consistency, we adopted the band gap value for the tetragonal phase. The single nucleus approximation in the Hamiltonian is justified because only the Ti atom (*M* = 47.867 g/mol) moves in the unit cell (*N* = 1), also with an electron number *N*_e_ = 1. The electron–phonon coupling parameter (*V* = 1.63 eV/Å) is provided by Cardona and coworkers.^22^ Utilizing these parameters in eq 25, we estimate the PTT to be 521 K. However, we emphasize a weak dependence (sensitivity) of the electron–phonon coupling parameter on the temperature. Existing literature reports a PTT of 400 K,^21^ indicating the significance of further investigation and refinement in this area.

We note that the main objective of the presented approach is not to provide a precise estimation of the PTT but to explain which terms in a single Hamiltonian result in the realization of different phases. For more precise values, it is necessary to employ numerical calculations that incorporate anharmonic phonon contributions, perhaps higher-order EPI contributions, as well as additional external perturbations. These contributions are obtained through extensive molecular dynamic simulations or density functional theory approaches. Examples are provided in the work of Toyoura et al.,^23^ Hellman et al.,^24^ and Bottin et al.^25^

## 4 Summary

This study models first-order solid–solid phase transitions by incorporating EPI and treating the kinetic energy of the nucleus on a QM basis. The temperature-dependent impact on the free energies is discussed, and the free energy is evaluated for two analytically solvable situations using the Bogoliubov inequality. The result predicts a first-order phase transition, with a HTP and a LTP arising due to the strong variation of the EPI contribution to the free energy with temperature. The temperature at which a phase transition occurs, the discrete shift of the nucleus, and the amount of latent heat released can be predicted without specifying crystallographic phases. Our findings, combined with previously published research on the same model system, narrow the temperature range and parameter space for first-order phase transitions.

## Supporting Information Available

Comparison of high temperature limit with classic case (PDF)
