# Supplementary material for: Prediction of First-Order Phase Transition with Electron–Phonon Interaction
Source: J Phys Chem C Nanomater Interfaces. 2024 Jun 11;128(24):10133–8. doi: 10.1021/acs.jpcc.4c00958 (PMC11194806; doi:10.1021/acs.jpcc.4c00958)
Supplement: Supplementary file 1 — jp4c00958_si_001.pdf [file jp4c00958_si_001.pdf]

Prediction of First-Order Phase-Transition with Electron-Phonon Interaction

Mario Graml[*,†,‡] and Kurt Hingerl[†]

†*Center for Surface- and Nanoanalytics - Johannes Kepler Universität, Altenbergerstr. 69, A-4040 Linz, Austria*

‡ *School of Education - Johannes Kepler Universität, Altenbergerstr. 69, A-4040 Linz, Austria*

E-mail: mario.graml@jku.at

High Temperature Limit

In order to validate eq. (21), it is necessary to examine its behaviour in the classical limit, corresponding to $\beta \to 0$. By utilizing eq. (21), we can determine the classical limit as follows:

$$F_{\mathrm{Mod}}^{(\mathrm{HTP})} \sim -\frac{1}{\beta}\ln\left(\frac{s(\beta)}{\beta\omega\hbar}\right) - \frac{V^2 N_{\mathrm{e}}^2}{2M\omega^2 N} + N_{\mathrm{e}}\frac{\varepsilon_1+\varepsilon_2}{2} \quad \text{as} \quad \beta \to 0. \tag{A.1}$$

To establish a comparison with the classical system, we transform eq. (4) into its classical representation, denoted as H_{C}. In this classical form, the electrons are considered nonentangled. This leads to separate solutions for the electronic, phonon, and electron-phonon Hamiltonian.:

$$H_{\mathrm{C}} = N_1\varepsilon_1 + (N_{\mathrm{e}} - N_1)\varepsilon_2 + \frac{1}{2}\left(\frac{P^2}{M} + M\omega^2 x^2\right) + (2N_1' - N_{\mathrm{e}})\frac{V}{\sqrt{N}}x. \tag{A.2}$$

Taking into account the multiplicity of N_{e} electrons with two bands, we calculate the classical partition function, denoted as Z_{C}, as follows:

$$Z_{\mathrm{C}} = \int_{-\infty}^{\infty}\int_{-\infty}^{\infty} \frac{\sum_{N_1=0}^{N_{\mathrm{e}}}\binom{N_{\mathrm{e}}}{N_1}\sum_{N_1'=0}^{N_{\mathrm{e}}}\binom{N_{\mathrm{e}}}{N_1'}\,\mathrm{e}^{-\beta H_{\mathrm{C}}}}{2\pi\hbar}\,\mathrm{d}x\,\mathrm{d}P. \tag{A.3}$$

Using Z_{C}, we can calculate the classical Helmholtz energy F_{C} as

$$F_{\mathrm{C}} = -\frac{1}{\beta}\ln\left(\frac{s(\beta)}{\beta\omega\hbar}\right) - \frac{V^2 N_{\mathrm{e}}^2}{2M\omega^2 N} + N_{\mathrm{e}}\frac{\varepsilon_1+\varepsilon_2}{2}. \tag{A.4}$$

It is evident that eq. (A.4) is equivalent to equation (A.1).
